# Model-informed drug development of envafolimab, a subcutaneously injectable PD-L1 antibody, in patients with advanced solid tumors

**DOI:** 10.1093/oncolo/oyae102

**Published:** 2024-07-09

**Authors:** Cheng Cui, Jing Wang, Chunyang Wang, Ting Xu, Lan Qin, Shen Xiao, John Gong, Ling Song, Dongyang Liu

**Affiliations:** Drug Clinical Trial Center, Peking University Third Hospital, Beijing, People’s Republic of China; Institute of Medical Innovation and Research, Peking University Third Hospital, Beijing, People’s Republic of China; Drug Clinical Trial Center, Peking University Third Hospital, Beijing, People’s Republic of China; Institute of Medical Innovation and Research, Peking University Third Hospital, Beijing, People’s Republic of China; Drug Clinical Trial Center, Peking University Third Hospital, Beijing, People’s Republic of China; Institute of Medical Innovation and Research, Peking University Third Hospital, Beijing, People’s Republic of China; Alphamab Co., Ltd., Suzhou, People’s Republic of China; 3DMedicines Co., Ltd., Shanghai, People’s Republic of China; 3DMedicines Co., Ltd., Shanghai, People’s Republic of China; 3DMedicines Co., Ltd., Shanghai, People’s Republic of China; Drug Clinical Trial Center, Peking University Third Hospital, Beijing, People’s Republic of China; Institute of Medical Innovation and Research, Peking University Third Hospital, Beijing, People’s Republic of China; Drug Clinical Trial Center, Peking University Third Hospital, Beijing, People’s Republic of China; Institute of Medical Innovation and Research, Peking University Third Hospital, Beijing, People’s Republic of China

**Keywords:** envafolimab, PD-L1 antibody, subcutaneous injection, population pharmacokinetics, exposure-response analysis

## Abstract

**Background and Objectives:**

Envafolimab is the first and only globally approved subcutaneously injectable PD-L1 antibody for the treatment of instability-high (MSI-H) or DNA mismatch repair deficient (dMMR) advanced solid tumors in adults, including those with advanced colorectal cancer that has progressed after treatment with a fluoropyrimidine, oxaliplatin, and irinotecan. The aim of this investigation was to examine the pharmacokinetic and exposure-response (E-R) profile of envafolimab in patients with solid tumors to support the approval of fixed and alternative dose regimens.

**Methods:**

In this study, a population pharmacokinetic (PopPK) modeling approach will be employed to quantitatively evaluate intrinsic and extrinsic covariates. Additionally, PopPK-estimated exposure parameters were used to evaluate E-R relationship for safety and efficacy to provide a theoretical basis for recommending optimal treatment regimens. Simulations were performed on the dosing regimens of body weight-based regimen of 2.50 mg/kg QW, fixed dose 150 mg QW, and 300 mg Q2W for the selection of alternative dosing regimens. Data from 4 clinical studies (NCT02827968, NCT03101488, NCT03248843, and NCT03667170) were utilized.

**Results:**

The PopPK dataset comprised 182 patients with 1810 evaluable envafolimab concentration records. Finally, a one-compartment model incorporating first-order absorption, first-order linear elimination, and time-dependent elimination according to an *E*_max_ function was found to accurately describe the concentration-time data of envafolimab in patients with advanced solid tumors. Creatinine clearance and country were identified as statistically significant factors affecting clearance, but had limited clinical significance. A relative flat exposure-response relationship was observed between early measures of safety and efficacy to verify that no dose adjustment is required. Simulation results indicated that 2.50 mg/kg QW, 150 mg QW, and 300 mg Q2W regimen yield similar steady-state exposure.

**Conclusions:**

No statistically significant difference was observed between weight-based and fixed dose regimens. Model-based simulation supports the adoption of a 150 mg weekly or 300 mg biweekly dosing regimen of envafolimab in the solid tumor population, as these schedules effectively balance survival benefits and safety risks.

## Teaser

This study examined the pharmacokinetic and exposure-response profile of envafolimab in patients with solid tumors to support the approval of fixed and alternative dose regimens.

**Implications for practice**

Envafolimab is the first and only globally approved subcutaneously injectable PD-L1 antibody for the treatment of MSI-H or dMMR advanced solid tumors in adults. Modeling and simulation methods are required to support marketing approval based on the best benefit-risk ratio. As a subcutaneously injectable fusion protein, population pharmacokinetic modeling of Envafolimab showed that creatinine clearance and country were identified as statistically significant factors affecting clearance with limited clinical significance. The model-based simulation provides support for the utilization of a 150 mg weekly or 300 mg biweekly dosing regimen in Chinese solid tumor population. Further data from multinational studies are required to comprehensively elucidate racial disparities and provide global endorsement for marketing purposes.

## Introduction

T cells play a crucial role in the immune system by mediating diverse cellular immune responses against pathogens.^1^ Meanwhile, immune checkpoints modulate immunological equilibrium by negatively regulating T-cell-mediated immune responses to maintain self-tolerance and prevent autoimmunity. The interaction between programmed cell death protein 1 (PD-1), a major immune checkpoint protein mainly expressed on activated T cells, and programmed cell death protein-ligand 1 (PD-L1) results in the attenuation of T-cell proliferation, migration, and cytotoxicity capacity.^2^ However, numerous tumor cells exploit this mechanism by upregulating PD-L1 expression on their surface, thereby activating the PD-1/PD-L1 signaling pathway and evading T-cell-mediated immune responses.^3,4^ Therefore, blocking the interaction between PD-1 and PD-L1 could restore T-cell function and enhance anti-tumor immunity.^5^ PD-1/PD-L1 inhibitors have demonstrated remarkable clinical responses and have led to revolutionary advances in cancer immunotherapy.^6^

Drug innovations in dosage forms and routes of administration play a pivotal role in enhancing patient adherence and optimizing disease management. Although all currently available immune checkpoint inhibitors are administered intravenously (IV), studies have consistently reported a patient preference for subcutaneous (SC) drug administration.^7,8^ As patients achieve durable responses and long-term survival with these agents, the repetitive IV infusions in the clinic may reduce work productivity, personal time, and increase healthcare costs.^9-11^ Therefore, SC administration not only mitigates the burden in clinical settings but also reduces medicalizes resource allocation, and enhances overall patient experience and satisfaction.^11^

Envafolimab, also known as KN035, is a novel homodimeric fusion protein consisting of a humanized single-domain PD-L1 antibody derived from camels and a mutated fragment crystallizable (Fc) region of human immunoglobulin G1 (IgG1), which are covalently linked by interchain disulfide bonds.^12^ Envafolimab is the first and only globally approved subcutaneously injectable PD-L1 antibody for treating adult patients with microsatellite instability-high (MSI-H) or DNA mismatch repair deficient (dMMR) advanced solid tumors and is currently undergoing clinical development for the treatment of a variety of cancer types.^13^ The heavy-chain-only structure of envafolimab confers it with properties such as low molecular weight, high water solubility, excellent stability, strong and rapid tissue penetration, and fast tumor enrichment rate. These features facilitate subcutaneous administration with minimal medical resource utilization and cost, reduced patient suffering, improved treatment compliance and absence of infusion reactions.^13,14^

The pharmacokinetics (PK), safety, efficacy, and immunogenicity of envafolimab were thoroughly assessed in phase I studies involving subjects with locally advanced or metastatic solid tumors, as well as in a phase II study involving subjects with advanced dMMR or MSI-H solid tumors. These assessments have been extensively documented in previous reports.^12,14-16^ In summary, different doses, including 0.01-10 mg/kg, 150 mg, and 300 mg, and various dosing regimens, including QW, Q2W, and Q4W were evaluated across these studies. Over the investigated dose range, the exposure of envafolimab was dose proportional. The median t_1/2_ of envafolimab was longer after multiple doses than after a single dose (23 days vs 14 days). Envafolimab was well tolerated, had durable antitumor activity, and acceptable safety after SC administration at a wide dose range and schedules.

In order to investigate the PK profile of envafolimab in the patient population with solid tumors, and quantitatively evaluate intrinsic and extrinsic factors that affect the in vivo distribution and disposal or relevant parameters of envafolimab. The population pharmacokinetics (PopPK) model was developed combining 3 phase I studies, which provided PK data from multiple doses with intensive sampling and facilitated structural model development, and one phase II study, which provided sparse sampling from a large population and facilitated covariate analysis. Moreover, exposure-response (E-R) analysis was further conducted to define the drug exposure-safety/efficacy relationship to provide a theoretical basis for the determination of drug therapeutic window and the design of recommended treatment regimens. The comprehensive PopPK and E-R analysis results reported herein supported the appropriateness and the approval of the clinical regimen 150 mg QW and 300 mg Q2W.

## Methods

### Participants and study design

The dataset used in this research comes from patients enrolled in 3 phase I studies, ie, KN035-US-001 (NCT02827968), KN035-CN-001 (NCT03101488), KN035-JP-001 (NCT03248843), and one phase II study KN035-CN-006 (NCT03667170). Four studies were conducted according to the Declaration of Helsinki and Good Clinical Practice and were approved by the institutional review boards or ethics committee at each site. Written informed consent and details of the trial were obtained from each patient before enrollment. A summary of the study treatments, patient population, PK sampling, and anti-drug antibody (ADA) sampling for each study included in this research is shown in Supplementary Table S1.

### PopPK model characterization

Plasma concentration data of envafolimab from patients with advanced solid tumors were analyzed using nonlinear mixed effects modeling approach implemented in the NONMEM program (Version 7.2; ICON Development Solutions, Hanover, MD, USA) called by Pirana software (Version 2.8.0). The estimation of parameters, variability, as well as the screening of covariate, were performed using Perl-Speaks-NONMEM (PsN, Version 4.8.1 or higher; Uppsala University, Uppsala, Sweden). Data management, exploratory data analysis (EDA), and graphical exploration were conducted in R software (Version 3.5.3; R Foundation for Statistical Computing, Vienna, Austria). Several model parameter estimation methods were used at different stages of the model development, including first-order (FO), first-order conditional estimation (FOCE), and first-order conditional estimation with interaction (FOCEI), the last of which was conducted in the final model parameter estimation.

### Covariate analysis

Covariate screening identified potential intrinsic and extrinsic factors that significantly influence the PK of envafolimab using parameter-covariate plots, the test of significance for correlation coefficient, and a stepwise approach. Stepwise approach consists of forward addition, in which the covariates contributing to a significant decrease in objective function value (OFV) of 6.63 (*P* < .01, chi-squared distribution with 1 df) were added one at a time, and backward deletion, in which the covariates contributing to a significant increase in OFV of 10.83 (*P* < .001, chi-square distribution with 1 df) were retained in the model.

### Model evaluation

To fully evaluate the reliability and robustness of the model, multiple evaluation methods were used. Goodness of fit (GOF) was used to evaluate the fitted deviations of the model. The visual predictive check (VPC) was used to evaluate the consistency between the model predictions and the observed data. VPC performed 1000 simulations on the final model by Monte-Carlo simulation, and the results from the simulations were calculated and compared with the observations. The bootstrap method was used to verify the estimated precision of the model parameters. By sampling the original data 500 times with replacement, 500 new data sets were obtained and fitted to the final model.

### Model simulation

In order to further investigate whether body weight-based dose adjustment of envafolimab was required within the body weight range of this study, simulations were performed on the PK profile of the study populations receiving the dosing regimens of body weight-based regimen of 2.50 mg/kg QW, fixed dose 150 mg QW, 300 mg Q2W for 20 weeks.

Bayesian estimation of individual envafolimab PK parameters was used to compute exposure variables, ie, area under the concentration-time curve from time 0 to infinity in cycle 1 (AUC_inf,1_), based on the actual dose regimen for E-R analysis.

### E-R analysis

Distinct software was implemented to achieve optimal data presentation and analysis due to methodological disparities. E-R analysis was evaluated by SAS (Version 9.4, SAS Institute, Cary, NC) and R software (Version 3.5.3; R Foundation for Statistical Computing, Vienna, Austria). Empiric individual Bayesian estimates of PK parameters were generated using the final PopPK model. Exploratory data analysis based on the quartile method, logistic regression analysis, and survival analysis were used to assess the significance of exposure predictors.

The exposure variables used for the E-R analysis were: minimum concentration in cycle 1 (*C*_min,1_), the maximum concentration in cycle 1 (*C*_max,1_), the average concentration in cycle 1 (*C*_avg,1_), PopPK model derived AUC_inf,1_, average trough concentration in each cycle (*C*_min, overall_).

Efficacy measures were the results of efficacy assessments performed by the investigator using imaging assessment (CT or MRI) according to RECIST V1.1, including change in target lesion size, progressive disease (PD), stable disease (SD), partial response (PR), complete response (CR), objective response rate (ORR), disease control rate (DCR), and duration of response (DOR). The analysis was performed based on optimal overall response assessment and overall change in the target lesion size of each subject.

Safety measures were the incidence and severity of drug-related TEAEs and AE of clinical interest (AECI). AECI was defined as drug-related severe AEs (grade 3 or higher), drug-related serious adverse events (SAEs), and drug-related adverse events of special interest (AESI) whose incidence was greater than 2.5% in the overall population.

## Results

### Participant demographics and baseline characteristics

Due to the high-concentration predose and abnormal PK profile postdose, one patient in the KN035-JP-001 study may have had prior exposure to other PD-L1 inhibitors and was excluded from this research. A total of 1810 envafolimab concentrations in 182 patients across the 4 studies were included in the PopPK analysis. Descriptive statistics of their demographic and baseline characteristics are presented in Supplementary Table S2.

A total of 452 patients from the 4 studies were included in the E-R analysis, with a reported count of 1745 drug-related TEAEs. The demographic data of patients of each analysis item are summarized in Supplementary Supplementary Table S3.

### Final PopPK model result

A one-compartment model with first-order absorption, first-order linear, and time-dependent elimination according to an *E*_max_ function well described the concentration-time data of envafolimab in patients with advanced solid tumors. The base model is described with the following formula:


$$\begin{array}{l} \frac{dA_{a}}{dt}=-k_{a}\cdot A_{a} \end{array}$$ (1)

$$\begin{array}{l} \frac{V}{F}\frac{dC_{p}}{dt}=k_{a}\cdot A_{a}-\frac{CL}{F}\cdot e^{\left(E_{max}\cdot TIME)/(T_{50}+TIME\right)}\cdot C_{P},\;C_{p}(0)=0 \end{array}$$ (2)

where *C*_p_ refers to the drug concentration in the central compartment, *V*/*F* refers to the apparent volume of distribution of the drug in the central compartment, CL/*F* refers to the apparent systemic clearance of the drug, *K*_a_ refers to the absorption rate constant of drug, *A*_a_ refers to the amount of the drug in the absorption compartment, *E*_max_ refers to the maximum change in clearance rate, and *T*_50_ refers to the time for clearance to reduce by half.

The inter-individual variability (IIV) of PK parameters was described using an exponential model. The proportional error model was used to describe the residual. The equations are as follows:


$$\begin{array}{l} P_{ij}=\theta_{i}\times e^{\eta_{ij}} \end{array}$$ (3)

$$\begin{array}{l} Y_{obs}=Y_{pred}\times (1+\;\varepsilon \;) \end{array}$$ (4)

where *P*_*ij*_ refers to the *i*th parameter value of the *j*th individual, θ_*i*_ refers to the typical value of the *i*th parameter, η_*ij*_ refers to the inter-individual variability of the *i*th parameter of the *j*th individual which follows the normal distribution (0, ω^2^), *Y*_obs_ and *Y*_pred_ refer to the observed concentration value and the predicted concentration value, ε refers to residual which follows the normal distribution (0, σ^2^).

Based on the base model, the preliminary significantly correlated continuous covariates (*P* < .01, Pearson correlation coefficient test) and the categorical covariates were selected as potential covariates for the stepwise approach. After 3 rounds of forward addition and one round of backward deletion, 2 statistically significant (*P* < .001) covariates, CRCL and country were eventually identified. For the screening process of important covariates, see Supplementary Table S4.

The quantitative relationship of the effect of CRCL and country on CL/F are as follows:

Chinese patients:


$$\begin{array}{l} \frac{CL}{F}=\theta_{CL\;\;\;}\times {\left(\frac{CRCL}{95.14}\right)}^{0.475}\times e^{\frac{E_{max}\;\;\;\cdot \;\;\;TIME}{T_{50}+\;\;\;TIME}}\times e^{\eta_{CL}} \end{array}$$ (5)

American (including White and Black) patients:


$$\begin{array}{l} \frac{CL}{F}=\theta_{CL\;\;\;}\times {\left(\frac{CRCL}{95.14}\right)}^{0.475}\times 0.891\times e^{\frac{E_{max}\;\;\;\cdot \;\;\;TIME}{T_{50}+\;\;\;TIME}}\times e^{\eta_{CL}} \end{array}$$ (6)

Japanese patients:


$$\begin{array}{l} \frac{CL}{F}=\theta_{CL\;\;\;}\times {\left(\frac{CRCL}{95.14}\right)}^{0.475}\times 0.74\times e^{\frac{E_{max}\;\;\;\cdot \;\;\;TIME}{T_{50}+\;\;\;TIME}}\times e^{\eta_{CL}} \end{array}$$ (7)

where CL/*F* refers to the systemic clearance rate, θ_CL_ refers to the typical CL/*F* value of the population, CRCL refers to the creatinine clearance rate (mL/minute), and η_CL_ refers to the random effect of CL/F.

### PopPK model evaluation

**Figure 1.**
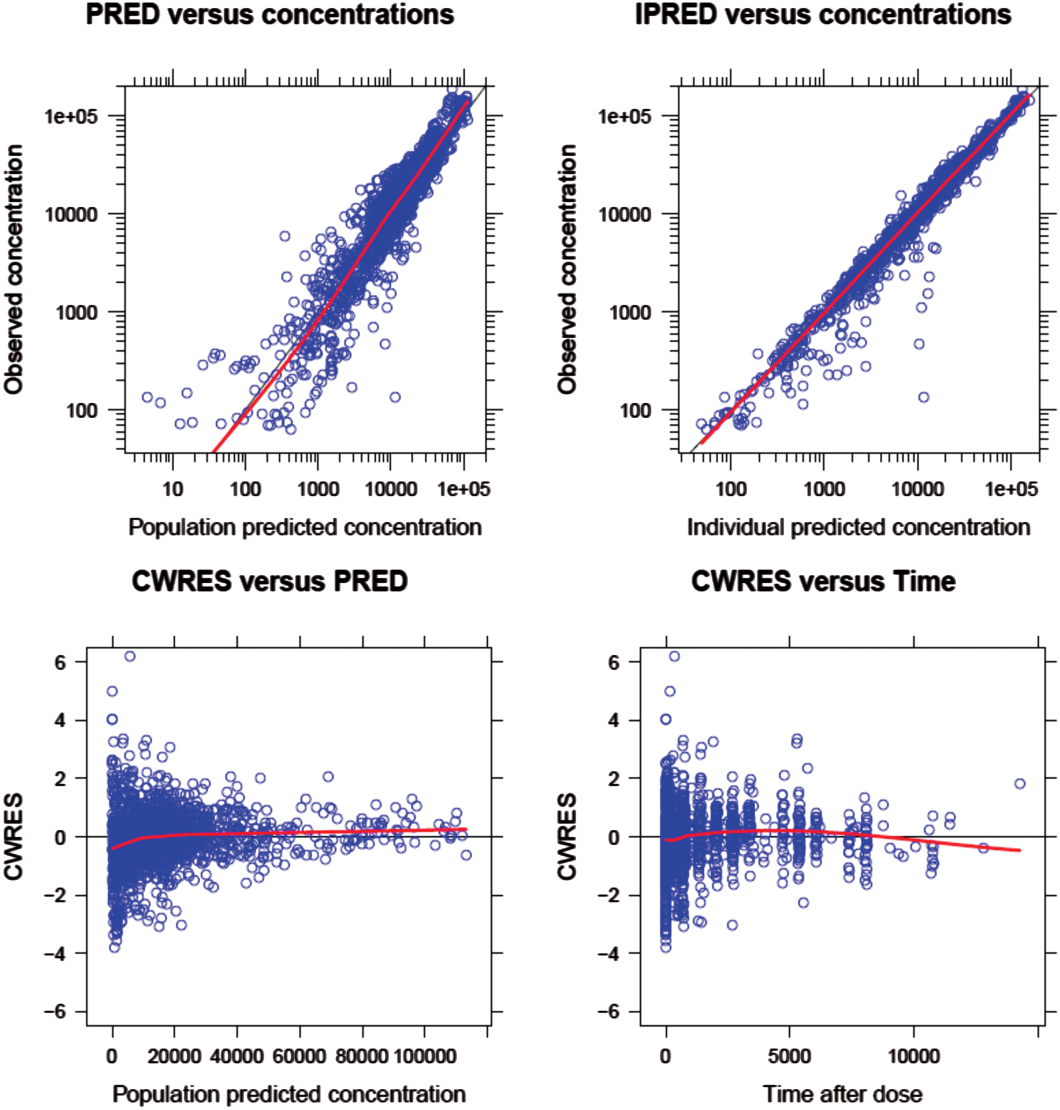
Goodness-of-fit (GOF) plots of the final PopPK model. The upper left of the GOF graph is the individual predicted concentration vs the observed concentration, the upper right graph is the population predicted concentration vs the observed concentration, the lower left graph is the conditional weighted residuals (CWRES) based on the FOCE algorithm vs the population predicted value, the lower right graph is CWRES vs. time after dose.

The parameter estimation of the final model is provided in Table 1. Compared with the base model, the IIV of CL/F reduced by approximately 6.9% (from 37.5% to 30.6%) after the addition of covariates, suggesting that the addition of covariates could partially explain the variability of parameters. The GOF plots of the final model, provided in Figure 1, proved that the final PopPK model could accurately describe the PK profile of envafolimab in Chinese, Japanese, and American patients with advanced or metastatic solid tumors.

**Table 1.** Parameter estimation of the final model and bootstrap result.

Parameter	Unit	Final model				Bootstrap result	
		Parameter estimate (RSE%)	95% CI	IIV% (RSE%)	Shrinkage (%)	Median	90% CI
*E*_max_	NA	−0.439 (14.1)	(−0.560, −0.318)	0 FIX (NA)	100	−0.464	(−0.727, −0.253)
*T*_50_	Day	34.0 (36.5)	(9.70, 58.3)	0 FIX (NA)	100	33.9	(13.8, 77.6)
CL	L/hr	0.0478 (7.9)	(0.0404, 0.0552)	30.6 (5.7)	13.8	0.0484	(0.0400, 0.0630)
*V*	L	15.5 (7.5)	(13.2, 17.8)	55.8 (6.4)	18.9	15.3	(13.6, 17.1)
*K*_a_	1/hr	0.0147 (8.4)	(0.0120, 0.0170)	60.9 (10.3)	38.6	0.0146	(0.0125, 0.0171)
CL-CRCL	NA	0.475 (19.3)	(0.295, 0.655)	NA (NA)	NA	0.474	(0.316, 0.596)
CL-AMERICAN	NA	0.891 (6.5)	(0.777, 1.01)	NA (NA)	NA	0.896	(0.739, 1.09)
CL-JAPANESE	NA	0.740 (8.0)	(0.624, 0.856)	NA (NA)	NA	0.740	(0.661, 0.827)
Epsilon	NA	0.0501 (1.8)	NA	NA (NA)	9.20	0.0496	(0.0418, 0.0604)

Abbreviations: CI: CI; NA: not applicable; IIV: inter-individual variability; *E*_max_: maximum change in clearance rate; *T*_50_: time for clearance rate to reduce by half; CL: systemic clearance of drug; *V*: volume of distribution; *K*_a_: absorption rate constant; CL-CRCL: effect of CRCL as a covariate on CL/F; CL-AMERICAN: effect of country as a covariate on CL/F (American vs. Chinese); CL-JAPANESE: effect of country as a covariate on CL/F (Japanese vs. Chinese); epsilon: model variation.

The internal validation performed using the bootstrap method showed that the convergence rate was 89.0%, the median of 500 bootstrap results was close to the final model parameter estimate, and the 90% CI included the final model parameter estimate, indicating the robustness of the established model.

**Figure 2.**
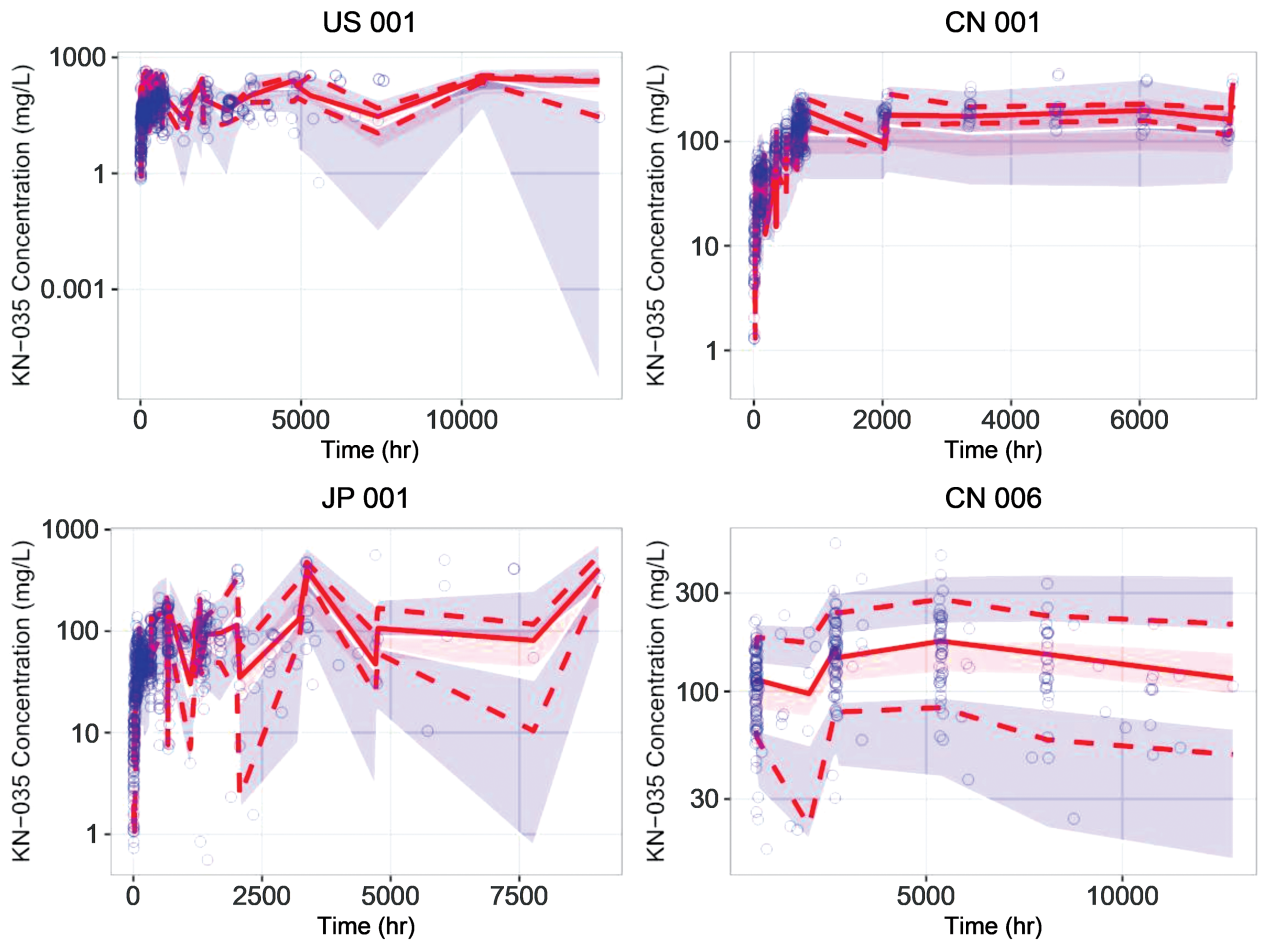
Dose-corrected visual predictive check (VPC) plots for the final PopPK model. Blue hollow dot: observations; solid and dotted red lines: the 5th, 50th, and 95th percentiles of the observations; red shadow: 95% CI of the 50th percentile of simulations; blue shadow: 95% CI of the 5th and 95th percentiles of simulations.

The dose-corrected VPC plots of the 4 studies are shown in Figure 2, from which there was no systematic bias between the estimations and the observations, suggesting that the precision of the final model was good, the model structure was acceptable, and could be used to accurately analyze the source of PK variability and the degree of its effects of envafolimab in Chinese, Japanese, and American patients with advanced or metastatic solid tumors.

### Weight-incorporated PopPK model

After covariate screening, WT was not retained as a significant covariate. To explore the need for body weight-based dose adjustment of envafolimab within the WT range of this study, WT was included in the final model as covariates for CL/*F* and V/*F* to develop a weight-incorporated PopPK model, resulting in a decrease of 7.742 in OFV compared to that of the final model.

The quantitative relationship of the effect of WT, CRCL, and country on CL/F and *V*/*F* are as follows:


$$\begin{array}{l} \frac{V}{F}=\theta_{V}\times {\left(\frac{WT}{63}\right)}^{0.623}\times e^{\eta_{V}} \end{array}$$ (8)

Chinese patients:


$$\begin{array}{l} \frac{CL}{F}=\theta_{CL\;\;\;}\times {\left(\frac{CRCL}{95.14}\right)}^{0.412}\times {\left(\frac{WT}{63}\right)}^{0.155}\times e^{\frac{E_{max}\;\;\;\cdot \;\;\;TIME}{T_{50}+\;\;\;TIME}}\times e^{\eta_{CL}} \end{array}$$ (9)

American (including White and Black) patients:


$$\begin{array}{l} \frac{CL}{F}=\theta_{CL\;\;\;}\times {\left(\frac{CRCL}{95.14}\right)}^{0.412}\times {\left(\frac{WT}{63}\right)}^{0.155}\times 0.842\times e^{\frac{E_{max}\;\;\;\cdot TIME}{T_{50}+\;\;\;TIME}}\times e^{\eta_{CL}} \end{array}$$ (10)

Japanese patients:


$$\begin{array}{l} \frac{CL}{F}=\theta_{CL\;\;\;}\times {\left(\frac{CRCL}{95.14}\right)}^{0.412}\times {\left(\frac{WT}{63}\right)}^{0.155}\;\times 0.73\times e^{\frac{E_{max}\;\;\;\cdot \;\;\;TIME}{T_{50}+\;\;\;TIME}}\times e^{\eta_{CL}} \end{array}$$ (11)

where CL/*F* refers to the apparent systemic clearance rate, *V*/*F* refers to the apparent volume of distribution, θ_CL_ refers to the typical CL/*F* value of the population, θ_V_ refers to the typical *V*/*F* value of the population, CRCL refers to the creatinine clearance rate (mL/minute), WT refers to body weight (kg), and η_CL_ refers to the random effect of CL/F, η_V_ refers to the random effect of V/F.

### Impact of covariates on envafolimab exposures

Based on the weight-incorporated PopPK model, the steady-state exposures of 150 mg QW envafolimab administered for 20 weeks on the typical population with different levels of covariates were simulated and compared to describe the impact of covariates on envafolimab exposures. Typical values of covariates of the simulation population are Chinese patients (typical WT 60.0 kg), American patients (typical WT 80.0 kg), and Japanese patients (typical WT 60.0 kg); normal renal function (CRCLCG > 90 mL/minute, the typical value calculated as 95.0 mL/minute), mild renal injury (90 mL/minute ≥ CRCLCG > 60 mL/minute, the typical value calculated as mean 75 mL/minute), and moderate renal injury (60 mL/minute ≥ CRCLCG > 30 mL/minute, the typical value calculated as the mean 45.0 mL/minute). None of the covariate extremes (maximum or minimum) had an effect greater than 50% of the median.

### Comparison of different dosing regimens

**Figure 3.**
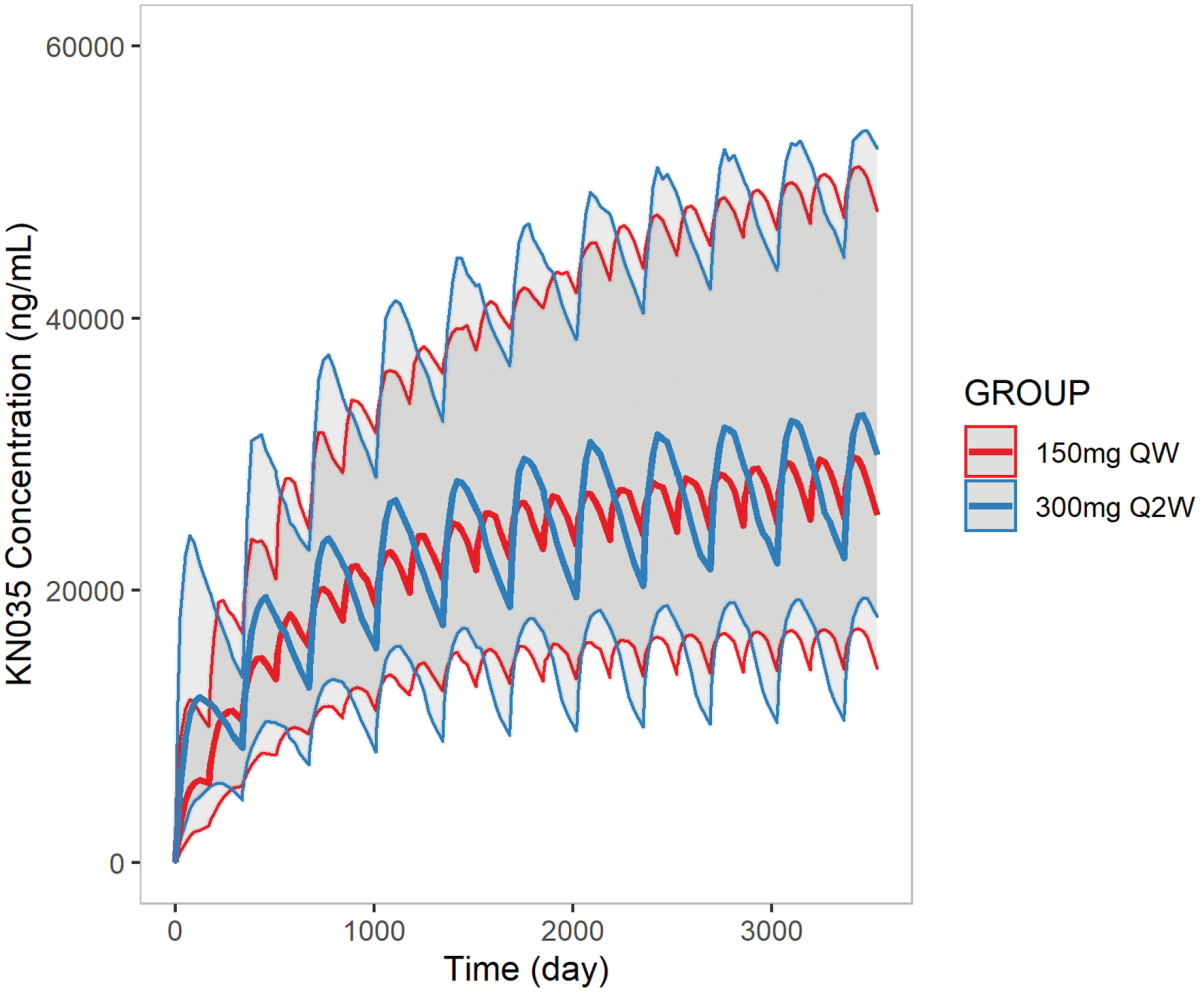
Simulated concentration-time curves of different dosing regimens after 20 weeks. Color bold lines: Median of simulated drug concentration; color thin lines: 90% CI of the median of simulated drug concentration; red: 300 mg Q2W; blue: 150 mg QW.

The concentration-time curves for 150 mg QW, 300 mg Q2W comparison were plotted in Figure 3, and the steady-state exposure parameters were summarized in Supplementary Table S5. Simulation results indicated that 150 mg QW, 2.50 mg/kg QW, and 300 mg Q2W regimen yield similar steady-state exposure in the overall population with the difference of parameters less than 15%.

The accumulation ratio of the different regimens is shown in Table 2, exhibiting that the longer the dosing interval, the lower the accumulation ratio. 150 mg QW and 2.50 mg/kg QW regimens yield obviously higher accumulation ratio than 300 mg Q2W regimen.

**Table 2.** Simulated accumulation ratio of different dosing regimens and proportion of patients with *C*_min,ss_ over 5.00 mg/L of different dosing regimens.

Dosing regimen	*R*_Cmax_			*R*_AUC_			Proportion, %
	Mean	SD	CV%	Mean	SD	CV%	
2.5 mg/kg QW	4.50	0.703	15.6	5.63	1.03	18.3	100
150 mg QW	4.50	0.703	15.6	5.63	1.03	18.3	100
300 mg Q2W	2.45	0.343	14.0	2.65	0.392	14.8	100

### Exposure-efficacy analysis

Among the 452 subjects administered envafolimab, 284 subjects had data on overall response assessment, 278 subjects had data on overall changes in target lesion size during the trial, and 76 subjects had DOR data.

**Figure 4.**
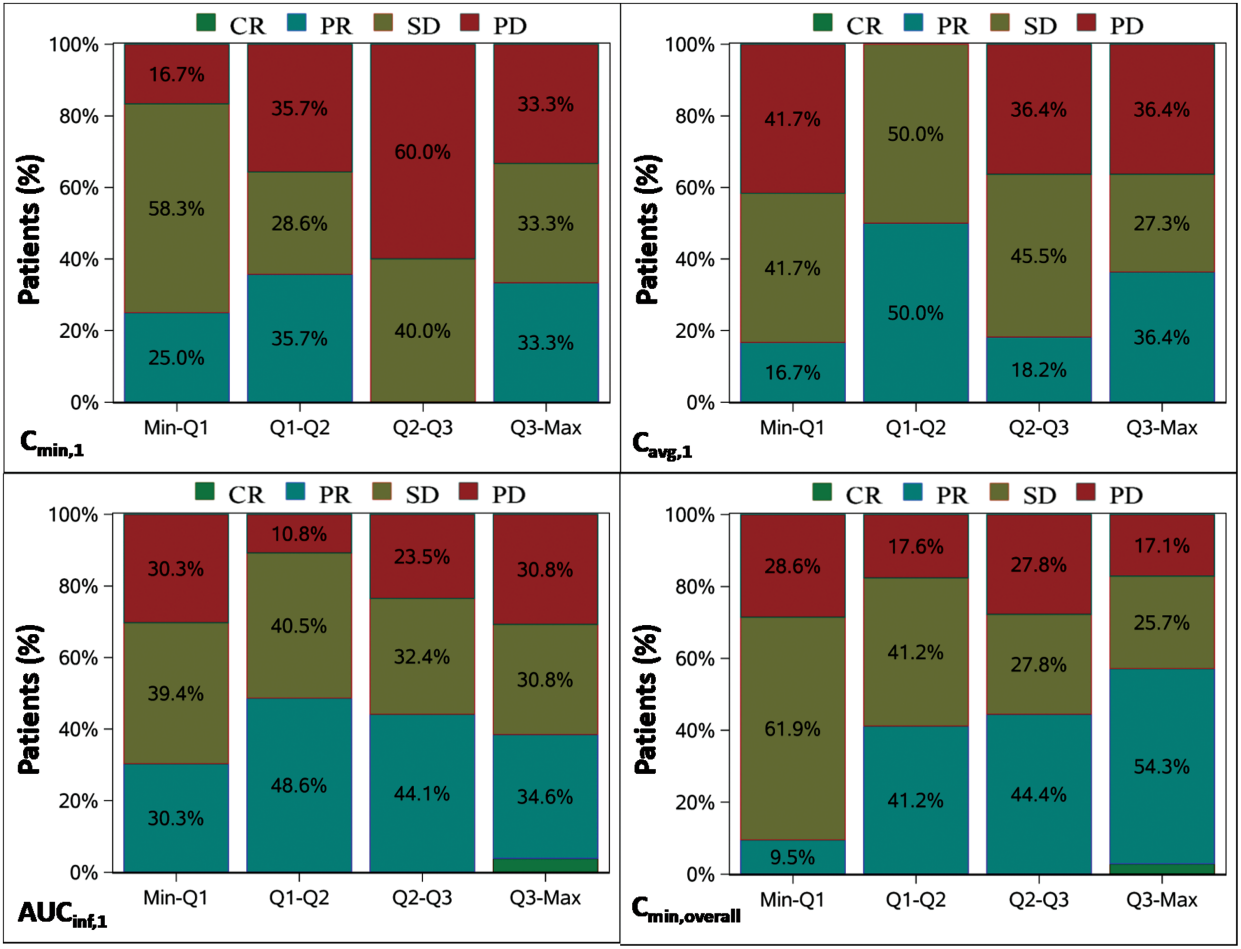
Optimal overall response across the quartiles of C_min,1_, C_avg,1_, AUC_inf,1_, and C_min,overall_.

As shown in Figure 4, no obvious correlation between *C*_min,1_, *C*_avg,1_, AUC_inf,1_ and optimal overall response was observed. With the increase of *C*_min,overall_, although DCR did not change significantly, the proportion of subjects with PR increased. Also, no correlation between *C*_min,1_, *C*_avg,1_, AUC_inf,1_ and overall changes in target lesion size was observed as seen in Supplementary Figure S1. However, with the increase of *C*_min, overall_, the overall changes in target lesion size were greater in the 2nd, 3rd, and 4th quartiles than in the 1st quartile, and there was no significant difference between the 2nd, 3rd, and 4th quartiles.

Kaplan-Meier curves for DOR were generated by median stratified exposure metrics of *C*_min,1_, *C*_avg,1_, AUC_inf,1_, and *C*_min,overall_, and results were presented in Supplementary Figure S2. With the increase of exposure metrics of *C*_min,1_, *C*_avg,1_, AUC_inf,1_, and *C*_min,overall_, no correlation between exposure and DOR was observed.

### Exposure-safety analysis

AECI categories included: increased AST (16.2%), increased ALT (14.6%), increased blood bilirubin (12.2%), rash (11.7%), hypothyroidism (10.4%), anemia (8.4%), hyperthyroidism (5.5%), fever (4.4%), diarrhea (4.0%), injection site reactions (4.0%), proteinuria (4.0%), abnormal hepatic function (3.3%), increased blood thyroid-stimulating hormone (3.1%), hyponatremia (2.7%), and pruritus (2.7%).

**Figure 5.**
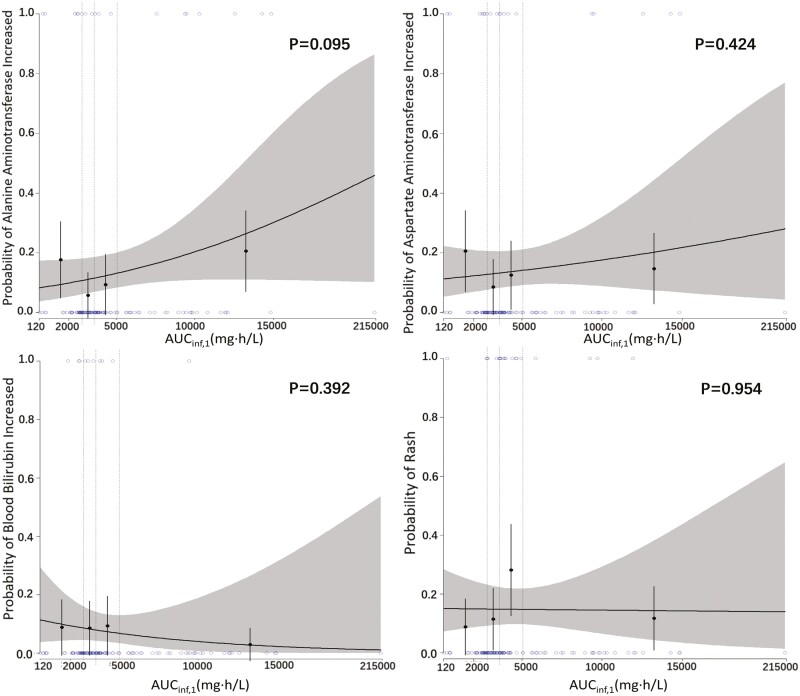
Distribution of probability incidence of top 4 AECI (increased AST, increased ALT, increased blood bilirubin, and rash) across AUC_inf,1_ quartile.

The AUC_inf,1_-safety analysis was conducted in 182 subjects with available AUC_inf,1_, where 15 AECIs were reported. The distribution of incidence and severity of the top 4 AECIs (increased AST, increased ALT, increased blood bilirubin, and rash) across the quartiles of AUC_inf,1_ were presented in Figure 5. No correlation between AUC_inf,1_ and the incidence and severity of AECI was observed. The *C*_max,1_-safety analysis was conducted in 79 subjects with available *C*_max,1_, where 12 AECIs were reported. The distribution of incidence and severity of the top 4 AECIs (increased AST, increased ALT, increased blood bilirubin, and rash) across the quartiles of *C*_max,1_ were presented in Supplementary Figure S3. No correlation between *C*_max,1_ and the incidence and severity of AECI was observed.

## Discussion

Antibody-mediated immune checkpoint blockade therapy has yielded breakthroughs in the field of cancer immunotherapies. Pharmacometrics play a vital role in the dose selection of this novel class of immune checkpoint inhibitors, which are tremendously different from that of traditional cytotoxic therapies based on the maximum tolerated dose (MTD) paradigm.^17-19^ The present analysis reports the population pharmacokinetic and exposure-response research of envafolimab in patients for the first time, which is the first and only globally approved subcutaneously injectable PD-L1 antibody for the treatment of MSI-H or dMMR advanced solid tumors in adults.

In the study to demonstrate the feasibility of SC administration of a PD-L1 inhibitor, a one-compartment model with first-order absorption, first-order linear, and time-dependent elimination according to an *E*_max_ function well described the concentration-time data of envafolimab in patients with advanced solid tumors. The liner and time-dependent clearance model was eventually used for model-based analysis in this study. The model showed that the CL/F of envafolimab decreased with the treatment time, and the time for clearance to reduce by half was 34 days. According to the EDA results and the previously reported PopPK models of formerly approved PD-1/PD-L1 inhibitors, the base model was tested as one- or 2-compartment models, linear elimination model or nonlinear elimination model, models with no change in drug clearance or with a time-dependent drug clearance, additional, proportional, or mixed error model.^20-22^

A recently published PopPK model of nivolumab consisted of 2 compartments, with zero-order infusion into the central compartment when administered by the i.v. route, first-order absorption from the extravascular compartment when administered via the s.c. route. Analysis showed that the estimated bioavailability (*F*) is 76.7%, and administration of rHuPH20 with nivolumab s.c. enabled considerably faster absorption compared with administration of nivolumab s.c. without rHuPH20. No other covariates were identified to have a clinically meaningful impact on either *K*_a_ or *F*.^23^ Preclinical pharmacokinetic studies in cynomolgus monkeys demonstrated complete drug absorption with a 104% bioavailability for envafolimab subcutaneous injection. However, no clinical studies were conducted on intravenous envafolimab. Due to significant interspecies variation in the bioavailability of monoclonal antibodies (mAbs) administered subcutaneously, predicting the human bioavailability of mAbs under development poses a considerable challenge.^24,25^

In contrast to conventional intravenous PD-1/PD-L1 antibodies, envafolimab has a unique structure without light chains and is half the molecular weight. This special design allows for better penetration into tumor tissue, improved stability at room temperature, and increased solubility in water to achieve higher concentrations needed for subcutaneous injection. These factors may explain the higher V estimates observed in this study compared to those reported for intravenous PD-1/PD-L1 antibodies. Additionally, a study on 10 subjects receiving envafolimab at a dosage of 300 mg every 4 weeks (US—001) found that after full administration of the dosage, PK samples were collected from 9 subjects (including necessary samples for phase analysis elimination). The apparent volume distribution was determined to be 18.7 L with a CV of 43.6%, which closely matched the model estimate of 15.5L.

The time-dependent changes were observed in most of the approved PD-1/PD-L1 inhibitors, while several mechanism hypotheses were proposed. Firstly, cachexia syndrome increased protein catabolism in advanced tumor patients, who consume the drugs as a source of protein in the case of metabolic imbalance, thus facilitating the degradation of those drugs. In the case of effective treatment, the CL/F decreases over time with the regression of the disease. In addition, some cancer cells can produce protease to cleave antibodies to evade host immune surveillance. Furthermore, changes in the endogenous protein turnover over time, assumed to be caused by a chronic inflammatory status in tumor patients, due to either the natural progression of the disease or the therapeutic effect of the drugs would then result in time-varying CL. Moreover, target-mediated drug disposition (TMDD), usually responsible for non-linear pharmacokinetics, could also play a role.^26-28^ To minimize the impact of response effects on CL/F and identify the causal E-R relationship, approaches such as the utilization of early exposures/baseline CL/F, ie, exposures after the first dose or first cycle, rather than steady-state exposures, have been used in the E-R analysis.

A significant association between CRCL and CL/*F* was observed. Based on the simulation of the weight-incorporated PopPK model, AUC_ss_, *C*_max,s_, and *C*_min,ss_ of patients with mild renal injury elevated by 9.9%, 9.6%, and 10.5%, respectively, compared with that of patients with normal renal function. AUC_ss_, *C*_max,ss_, and *C*_min,ss_ of patients with moderate renal injury elevated by 34.4%, 33.3%, and 36.3%, respectively, compared with that of patients with normal renal function. The Fc fusion protein envafolimab is expected to be broken down into peptides and amino acids in the body, which are then excreted or reused for protein or peptide synthesis. Biodistribution of ^89^Zr-envafolimab in cynomophagous monkeys also showed that there were no significant radioactive substances in other major organs except liver and kidney, which may be related to the liver and kidney as the main metabolic organs of drugs. Wu et al reported that the molecular weight of fusion proteins enable renal filtration and excretion and that the clearance of trebananib, a 64 kDa peptide-Fc fusion protein, decreased with diminishing estimated glomerular filtration rate (eGRF).^29^ Brandl et al investigated the pharmacokinetics of several fusion proteins ranging from 19 to 99 kDa and found the kidney to be the major organ of excretion for all tested constructs.^30^Unlike full-length monoclonal antibodies, peptibodies can undergo renal filtration and excretion. The molecular weight of envafolimab was only slightly more than that of albumin (79 kDa vs 69 kDa), making it possible for renal clearance. The inter-individual variation of CRCL was found to be 34.8%. Nevertheless, the subjects with moderate renal injury only accounted for 8.2% of the whole population of this study, so the estimated changes in PK parameters in patients with moderate renal injury could also be affected by sampling error, and the reliability of its application needs to be validated by including more patient data in the future. Although CRCL has a statistically significant effect on CL/F, the magnitude of the effect was relatively small. Compared with CRCL at the median (95.1 mL/minute), CRCLCG decreased by 16.1% at the 10th percentile (62.2 mL/minute) and increased by 18.9% at the 90th percentile (145.1 mL/minute). The changes were all less than 20.0%. Based on the flat E-R relationships, CRCL did not affect envafolimab exposure with clinical significance, and therefore no dose adjustment is required based on renal function.

The exposure of envafolimab was not significantly affected by the country, according to the E-R analysis results. The distribution of cancer types in the original dataset was as follows: colon cancer (35.7%), stomach cancer (9.8%), biliary tract cancer (6.0%), rectal cancer (5.5%), and urothelial cancer (4.4%). Since the proportion of other tumor species besides colon cancer was below 10%, it was not possible to draw a reliable conclusion from this analysis alone. Therefore, for this study, tumor types were categorized into 2 groups: colon cancer and non-colon cancer. The proportion of people with colon cancer in China (49%), the US (14%), and Japan (6%) followed a similar trend as the estimated value of CL/F in each country, suggesting a potential correlation between cancer and country. During covariate screening, both variables were evaluated separately, revealing that country had a greater impact with larger dOFV value. Therefore, countries were considered significant covariates in the final model. The difference in CL/F between Chinese and Japanese patients may be attributed to variations in tumor types and the small sample size. Despite race being included as a covariate in the final PopPK model and differences in exposure/response across racial groups being identified in recent studies, only a limited number have been translated into population-specific dose recommendations. Although gene/target protein expression, tumor burden, disease progression could play a part in the impact of race on the PK of PD-1/PD-L1 inhibitors, the exact mechanism remains to be studied.^31^ To better understand the pharmacokinetic characteristics and influential factors, the model will be further optimized with more data in the future.

A PopPK model here was developed using existing data to determine pharmacokinetic equivalence among 3 treatment regimens (2.5 mg/kg QW, 150 mg QW, and 300 mg Q2W). All regimens yield *C*_min,ss_ higher than the effective concentrations (11.5-18.7 mg/L vs 5.00 mg/L) required by the preclinical pharmacodynamics study, which would not show significant difference in drug efficacy.^2,15^ The current model did not include weight as a significant covariate, but it is likely to be associated with the sample size and relatively narrow range (90%CI: 47.7-92.8 kg). Pharmacokinetic simulations were conducted to evaluate the quantitative effects of body weight after based on the weight-incorporated PopPK model. For Chinese and Japanese patients with advanced or metastatic solid tumors, envafolimab exhibited similar steady-state exposure between the fixed dose regimen of 150 mg QW and the body weight-based regimen of 2.50 mg/kg, supporting that the fixed dose regimen of 150 mg QW can be used within the body weight range of (39.5, 120.0) kg. Nonetheless, for the American population, there was an approximately 37.0% difference in drug exposure of envafolimab between the fixed dose regimen of 150 mg QW and the body weight-based regimen of 2.50 mg/kg QW. This could be explained by the larger average body weight in American patients (average body weight 83.0 kg in the American patients in this study), whereas the fixed dose of 150 mg was converted based on 60 kg (typical body weight in the Chinese and Japanese populations). For Chinese, American, and Japanese patients with advanced or metastatic solid tumors, compared with the fixed dose regimen of 150 mg QW, the fixed dose regimen of 300 mg Q2W showed consistent total exposure and more obvious fluctuation in the peak-to-valley ratio. In addition, it should be noted that drug efficacy ranging from 0.3 to 10 mg/kg was observed in our clinical trials. Importantly, the sample sizes for the 2.5 mg/kg QW and 150 mg QW groups were significantly larger compared to other doses, offering more definitive evidence of effectiveness. We cannot definitively conclude that doses below 150 mg QW or 300 mg Q2W are necessarily inferior. Further analysis will incorporate additional clinical trial data and dosing regimens that are currently being explored.

A total of 452 patients participated in E-R analysis and received at least one dose of the drug. Referring to studies with other PD-1/PD-L1 inhibitors, in the case of effective treatment, the clearance of monoclonal antibodies decreases over time with the regression of the disease. Exposure metrics at steady state do not truly reflect the impact of drug exposure on efficacy considering the potentially confounding factors such as time-varying clearance. Hence, exposure metrics within the first cycle (*C*_min,1_, *C*_avg,1_, AUC_inf,1_) were more reasonable to reflect true E-R relationship than steady-state exposure metrics (*C*_min,overall_). Seventy-nine out of 452 patients underwent intensive sampling during the first cycle, yet no discernible correlation trend was observed between exposure metrics (*C*_max,1_, *C*_min,1_ and *C*_avg,1_) and safety or efficacy outcomes. Although further expansion of the sample size is necessary to explore this correlation, a flat and plateaued E-R relationship has been extensively reported in PD-1/PD-L1 inhibitors.^26,32,33^ The E-R analysis did not reveal any significant relationships, which can be attributed to 2 factors. Firstly, the sample size was imbalanced, with a significantly higher number of subjects in the medium-dose group compared to the low-dose and high-dose groups. Secondly, it is possible that target saturation occurs. In vitro studies showed that envafolimab had an average dissociation constant (*K*_d_) of 2.86 nM (226 ng/mL) for binding to human PD-L1, with concentration-dependent behavior and a higher binding activity than durvalumab (EC50 value of 62.275 ng/mL). Envafolimab effectively stimulated CD4^+^ T cells and induced IFN-γ release at concentrations between 0.002-0.2 μg/mL, with slightly stronger potency compared to durvalumab at higher concentrations. Based on these results, a clinical target concentration of 5 μg/mL was proposed. According to the PopPK simulation results, the minimum steady-state drug concentration will reach 5 μg/mL at 100 mg QW. Even with lower dose regimens, a different proportion of subjects will reach this target.

In this study, the most common drug-related adverse events (≥ 5%) of envafolimab in monotherapy were increased AST (16.2%), increased ALT (14.6%), increased blood bilirubin (12.2%), rash (11.7%), and hypothyroidism (10.4%). Although comparing AE incidence rates between different trials may not accurately reflect actual clinical practice, trends can be observed that the type and frequency of adverse events of envafolimab in monotherapy are similar to those of other PD-1/PD-L1 inhibitors, and the overall safety characteristics were comparable.^33-35^ Similar to the reported anti-PD-1 antibody PF-06801591, no significant injection-site toxic effects were observed following subcutaneous administration in envafolimab.^11^ Therefore, envafolimab in monotherapy has good safety and tolerability and is basically similar to other marketed anti-PD-1/PD-L1 antibodies, except that it is administered subcutaneously with no infusion reactions and a rare injection site reaction rate.

Improvement of drug dosing regimens, including transformation from body weight-based regimen to fixed dose regimen, less frequent treatment, and infusion times were supported by the application of PopPK and E-R analysis of approved anti-PD-1/PD-L1 antibodies. Those marketed drugs were either approved as body weight-based regimens and then reassessed and approved subsequently as fixed dose regimens, or approved initially as fixed dose regimens. The fixed dose was chosen based on the broad therapeutic index of this class of drugs and to ensure a significant rate of overlap between the exposure of fixed dose regimen and body weight-based regimen over the study body weight range, which offers practical benefits such as minimizing drug waste, reducing the risk of administration error, easier dose preparation, convenience to health care providers, improving compliance and patient adherence and reducing concerns regarding accurate dosing in patients with weight fluctuations.^17,36-38^

## Conclusion

In conclusion, this article describes the PK of envafolimab in the patient population with solid tumors using PopPK modeling approach and assessed the envafolimab E-R relationship for safety and efficacy. The model-based simulation supported the application of the 150 mg QW and 300 mg Q2W dosing regimen in adult patients with MSI-H or dMMR advanced solid tumors.

## Supplementary material

Supplementary material is available at *The Oncologist* online.

oyae102_suppl_Supplementary_Tables

oyae102_suppl_Supplementary_Figures

## Data Availability

The data for this study are available from the corresponding author upon reasonable request.
