# Supplementary material for: Model-informed drug development of envafolimab, a subcutaneously injectable PD-L1 antibody, in patients with advanced solid tumors
Source: Oncologist. 2024 Jul 9;29(9):e1189–200. doi: 10.1093/oncolo/oyae102 (PMC11379657; doi:10.1093/oncolo/oyae102)
Supplement: oyae102_suppl_Supplementary_Tables [file oyae102_suppl_supplementary_tables.docx]

**Model-informed Drug Development of Envafolimab,** **a Subcutaneously Injectable PD-L1 Antibody, in Patients with Advanced Solid Tumors**

Cheng Cui^1, 2, †^, Jing Wang^1, 2, †^, Chunyang Wang^1, 2^, Ting Xu^3^, Lan Qin^4^, Shen Xiao^4^, John Gong^4^, Ling Song^1, 2, *^, Dongyang Liu^1, 2, *^

^1^ Drug Clinical Trial Center, Peking University Third Hospital, Beijing, China.

^2^ Institute of Medical Innovation and Research, Peking University Third Hospital, Beijing, China.

^3^ Alphamab Co., Ltd., Suzhou, China.

^4^ 3DMedicines Co., Ltd., Shanghai, China.

^†^Cheng Cui and Jing Wang contributed equally to this work and share first authorship.

^*^ Correspondence：

🖂 Dongyang Liu, liudongyang@vip.sina.com.

Drug Clinical Trial Center, Peking University Third Hospital, Beijing, 100191, China.

Tel: (010) - 82266456

^*^Co-Correspondence:

🖂 Ling Song, sl_onging@sina.cn.

Drug Clinical Trial Center, Peking University Third Hospital, Beijing, 100191, China.

Tel: (010) - 82266455

Key words: Envafolimab, PD-L1 antibody, subcutaneously injection, population pharmacokinetics, exposure-Response Analysis,

**CONFLICT OF INTEREST**

The authors declared no competing interests for this work.

**FUNDING**

This research was supported by Bill and Melinda Gates Foundation (INV- 007625).

Supplementary Tables

[Table S1 Summary of Clinical Studies and Data Included in the PopPK and E-R Analysis 3](#_Toc140927222)

[Table S2 Descriptive statistics of demographic and baseline characteristics in PopPK analysis 5](#_Toc140927223)

[Table S3 Descriptive statistics of demographic in E-R analysis 6](#_Toc140927224)

[Table S4 Key Modeling Covariate Screening Procedures 7](#_Toc140927225)

[Table S5 Simulated steady-state exposures of different dosing regimens after 20 weeks 8](#_Toc140927226)

#

# Table S1 Summary of Clinical Studies and Data Included in the PopPK and E-R Analysis

| **Study (ClinicalTrials. gov identifier)** | **Phase** | | **Envafolimab treatment*** | **Population** | **N** | **PK sampling times** | **ADA sampling times** |
| --- | --- | --- | --- | --- | --- | --- | --- |
| KN035-US-001  (NCT02827968) | I | dose escalation  part | 0.01 mg/kg,  0.03 mg/kg,  0.1 mg/kg,  0.3 mg/kg,  1.0 mg/kg,  2.5 mg/kg,  5 mg/kg,  10 mg/kg,  QW | locally advanced or metastatic solid tumors | 18 | C1: Day 1 pre-dose and post-dose 6, 9, 24, 48, 96 hrs and pre-dose and post- dose 9 hrs on Day 8, 15 and 22.  C2 and Additional Cycles: Day 1 pre-dose and 9,hrs post-dose. | C1, C2, every other subsequent cycle: Day 1 pre-dose. |
|  |  | dose exploration  part | 300 mg,  Q4W |  | 10 | C1, C5: pre-dose; post-dose: 6, 24, 48, 72, 96, 168, 336, and 504 h.  C2 through C4: Day 1 pre-dose and Day 4 at 72 h post-dose.  C6 and subsequent cycles until C12: Day 1 pre-dose. | C1, C5: Day 1 pre-dose, D15 at 336 h post dose.  C2 and subsequent cycles until C12: Day 1 pre-dose. |
| KN035-CN-001  (NCT03101488) | I | dose escalation  part | 0.1 mg/kg,  0.3 mg/kg,  1.0 mg/kg,  2.5 mg/kg,  5 mg/kg,  10 mg/kg,  QW | locally advanced or metastatic solid tumors | 17 | C1: Day 1 pre-dose and post-dose 2, 4, 8, 12, 24, 48, 72, 96, 120 hrs and pre-dose and post- dose 8 hrs on Day 8, 15 and 22.  C2: Day 1 pre-dose and post-dose 2, 4, 8, 12, 24, 48, 72, 96, 120 hr.  C4, 6, 8, 10, 12: Day 1 pre-dose and 8 h post-dose. | C1, 2: Day 1 pre-dose.  C4, 6, 8, 10, 12: Day 1 pre-dose |
|  |  | dose exploration part-1 | 2.5 mg/kg, 5.0 mg/kg,  QW | advanced HCC | 40 | No PK sampling, but included in ER analysis. | No ADA sampling, but included in ER analysis. |
|  |  | dose exploration part-2 | 2.5 mg/kg, 5.0 mg/kg,  QW | locally advanced or metastatic solid tumors | 230 |  |  |
| KN035-JP-001  (NCT03248843) | I | dose escalation  part | 1.0 mg/kg,  2.5 mg/kg,  5 mg/kg,  10 mg/kg,  QW | locally advanced or metastatic solid tumors | 10 | C1: Day 1 pre-dose and post-dose 6, 9, 24, 48, 96,hrs and pre-dose and post- dose 9 hrs on Day 8, 15 and 22.  C2 and subsequently every other cycle during the first 6 cycles: Day 1 pre-dose and 9,hrs post-dose.  C8 and subsequently every other cycle until C12: Day 1pre-dose | C1, C2, every other subsequent cycle until C12: Day 1 pre-dose. |
|  |  | dose expansion part-1 | 2.5 mg/kg,  5 mg/kg,  Q2W |  | 16 | C1: Day 1 pre-dose and post-dose 6, 9, 24, 48, 72, 96, 120, 168, 216, 288 hrs and Day 15 pre-dose.  C2: Day 1 and Day 15 pre-dose.  C3: Day 1 pre-dose and post-dose 6, 9, 96 hrs and Day 15 pre-dose.  Additional Cycles: Day 1 of C4 and subsequently every other cycle until C12 at pre-dose. | C1, 2, 3, 4, every other subsequent cycle until C12: Day 1 pre-dose. |
|  |  | dose expansion part-2 | 300 mg,  Q4W |  | 9 | C1, C5: pre-dose; post-dose: 6, 24, 48, 72, 96, 168, 336, 504 h.  C2 and additional Cycles: Day 1 pre-dose and 72 h post-dose. | C1, C5: Day 1 pre-dose, D15 at 336 h post dose.  Every other subsequent cycle until C12: Day 1 pre-dose. |
| KN035-CN-006  (NCT03667170) | II | | 150 mg,  QW | advanced dMMR/MSI-H colorectal carcinoma or other advanced solid tumors | 103 | C1, 2, 5 and subsequently every four cycles: Day 1 pre-dose. | C1, 2, 5 and subsequently every four cycles: Day 1 pre-dose. |

* Administration routes were all subcutaneous injections.

# Table S2 Descriptive statistics of demographic and baseline characteristics in PopPK analysis

| **Continuous Covariate** | **Median** | **Mean** | **Range** |  |
| --- | --- | --- | --- | --- |
| Age, years | 55.0 | 54.9 | (22.0-79.0) |  |
| Body height, cm | 168 | 167 | (143-188) |  |
| Body weight, kg | 63.0 | 66.3 | (39.5-120) |  |
| Lean body weight, kg | 47.3 | 47.5 | (35.7-63.2) |  |
| Body surface area, m^2^ | 1.69 | 1.73 | (1.34-2.33) |  |
| Body mass index, kg/m^2^ | 23.3 | 23.6 | (14.9-40.7) |  |
| White blood cell count, *10^9^/L | 5.87 | 6.27 | (2.10-15.5) |  |
| Red blood cell count, *10^12^/L | 4.00 | 4.03 | (2.85-5.24) |  |
| Hemoglobin, g/L | 116 | 104 | (9.50-155) |  |
| Hematocrit, % | 35.9 | 32.5 | (0.306-48.0) |  |
| Platelet count, *10^9^/L | 230 | 237 | (85.0-567) |  |
| Alanine aminotransferase, U/L | 17.0 | 21.4 | (4.00-80.0) |  |
| Aspartate aminotransferase, U/L | 22.0 | 26.1 | (8.30-98.0) |  |
| Total protein, g/L | 72.0 | 71.9 | (57.0-88.8) |  |
| Albumin, g/L | 41.0 | 40.9 | (22.0-52.1) |  |
| Total bilirubin, μmol/L | 9.70 | 10.7 | (3.10-30.1) |  |
| Creatinine, μmol/L | 64.8 | 69.9 | (35.0-172) |  |
| Creatinine clearance (CRCLCG), mL/min | 95.1 | 98.8 | (36.6-282) |  |
| Glucose, mmol/L | 5.38 | 5.69 | (3.63-12.9) |  |
| Lactate dehydrogenase, U/L | 204 | 243 | (85.0-2320) |  |
| Urine specific gravity | 1.02 | 1.02 | (1.00-1.07) |  |
| Alkaline phosphatase, U/L | 99.5 | 165 | (31.0-1740) |  |
| Sum of target lesion diameters, mm | 61.0 | 76.8 | (10.0-296) |  |
| **Categorical covariate** | **Patients, n (%)** | | | |
| Sex |  | | | |
| Male | 115 (63.2) | | | |
| Female | 67 (36.8) | | | |
| Country |  | | | |
| China | 120 (65.9) | | | |
| USA | 28=26+2 (15.4) | | | |
| Japan | 34 (18.7) | | | |
| Tumor type |  | | | |
| Colon cancer | 65 (35.7) | | | |
| Other cancers | 117 (64.3) | | | |
| Concomitant use of  opioids |  | | | |
| Yes | 74 (40.7) | | | |
| No | 108 (59.3) | | | |
| Presence of ADA Positive |  | | | |
| Yes | 107 (58.8) | | | |
| No | 75 (41.2) | | | |
| Renal function |  | | | |
| Normal renal function  (CRCLCG > 90 mL/min) | 107 (58.8) | | | |
| Mild renal injury  (90 mL/min ≥ CRCLCG > 60 mL/min) | 60 (33) | | | |
| Moderate renal injury  (CRCLCG ≤ 60 mL/min) | 15 (8.2) | | | |

# Table S3 Descriptive statistics of demographic in E-R analysis

| **Characteristic** | | **Overall E-R analysis population**  **(N = 452)** | **C_max,1_, C_min,1_ and C_avg,1_ analysis population**  **(N = 79)** | **AUC_inf,1_ analysis population**  **(N = 182)** | **C_min,overall_ analysis population**  **(N = 168)** |
| --- | --- | --- | --- | --- | --- |
| Age  (year) | Mean (SD) | 54.18±12.18 | 58.77±13.22 | 54.92±13.51 | 55.20±13.45 |
|  | Median (CV %) | 55.00 (22.49%) | 62.00 (22.49%) | 55.00 (24.60%) | 55.50 (24.36%) |
|  | Range | (20.00-79.00) | (24.00-79.00) | (22.00-79.00） | (22.00-79.00） |
| BMI  (kg/m^2^) | Mean (SD) | 22.96±3.90 | 25.38±4.89 | 23.63±4.53 | 23.68±4.65 |
|  | Median (CV %) | 22.85 (17.00%) | 24.72 (19.26%) | 23.26 (19.19%) | 23.21 (19.63%) |
|  | Range | (14.20-40.70) | (16.56-40.70) | (14.87-40.70) | (14.87-40.70) |
| Sex | Male, N (%) | 293 (64.82%) | 50 (63.29%) | 115 (63.19%) | 105 (62.5%) |
|  | Female, N (%) | 159 (35.18%) | 29 (36.71%) | 67 (36.81%) | 63 (37.5%) |
| Study | KN035-CN-001, N (%) | 287 (63.50%) | 17 (21.52%) | 17 (9.34%) | 15 (8.93%) |
|  | KN035-CN-006, N (%) | 103 (22.79%) | NA | 103 (56.59%) | 95 (56.55%) |
|  | KN035-JP-001, N (%) | 34 (7.52%) | 34 (43.04%) | 34 (18.68%) | 31 (18.45%) |
|  | KN035-US-001, N (%) | 28 (6.19%) | 28 (35.44%) | 28 (15.39%) | 27 (16.07%) |

# Table S4 Key Modeling Covariate Screening Procedures

| **No.** | **Model Description** | **OFV** | **ΔOFV** | **Remarks** | |
| --- | --- | --- | --- | --- | --- |
| Base Model | | | | |  |
| 026 | One-compartment model with first-order absorption and first-order linear and time dependent elimination | 26786.51 |  | Base Model | |
| Forward Addition | | | | |  |
| 0011 | Effect of alkaline phosphatase on CL added to 026 | 26772.544 | -13.966 |  | |
| 0012 | Effect of CRSLSG on CL added to 026 | 26747.082 | -39.428 | Addition | |
| 0013 | Effect of tumor type on CL added to 026 | 26771.38 | -15.13 |  | |
| 0016 | Effect of Country on CL added to 026 | 26758.806 | -27.704 |  | |
| 0023 | Effect of CRCLCG on V added to 026 | 26771.688 | -14.822 |  | |
| 2012 | Effect of tumor type on CL added to 0012 | 26736.948 | -10.134 |  | |
| 2015 | Effect of Country on CL added to 0012 | 26730.03 | -17.052 | Addition | |
| 3020 | Effect of CRCLCG on V added to 2015 | 26720.088 | -9.942 | Addition, 3020 was the full model | |
| Backward deletion | | | | |  |
| 5001 | Effect of CRCLCG on CL excluded from 3020 | 26744.249 | +24.161 |  | |
| 5002 | Effect of Country on CL excluded from 3020 | 26737.414 | +17.326 |  | |
| 5003 | Effect of CRCLCG on V excluded from 3020 | 26730.03 | +9.942 | Deletion | |
| 6001 | Effect of CRCLCG on CL excluded from 5003 | 26758.803 | +28.773 | All retained, 5003 was the final model | |
| 6002 | Effect of Country on CL excluded from 5003 | 26747.082 | +17.052 |  |  |

# Table S5 Simulated steady-state exposures of different dosing regimens after 20 weeks

| **Dosing**  **Regimen** | **Population** | **AUC_τ,ss_** | | | | **C_max,ss_** | | | | **C_min,ss_** | | | |
| --- | --- | --- | --- | --- | --- | --- | --- | --- | --- | --- | --- | --- | --- |
|  |  | **Median**  **(*10^-6^ mg∙h/L)** | **SD** | **CV%** | **Range**  **(*10^-6^ mg∙h/L)** | **Median (mg/L)** | **SD** | **CV%** | **Range (mg/L)** | **Median (mg/L)** | **SD** | **CV%** | **Range (mg/L)** |
| 2.5 mg/kg QW | Overall | 4904 | 1391 | 26.0 | 3452 - 9565 | 30.3 | 8.33 | 25.3 | 21.5 - 58.1 | 27.1 | 8.16 | 27.3 | 18.7 - 54.6 |
|  | US | 7117 | 1031 | 14.6 | 5017 - 9414 | 43.5 | 6.20 | 14.3 | 30.9 - 57.4 | 40.3 | 6.00 | 15.0 | 27.9 - 53.5 |
|  | Non-US | 4716 | 1226 | 24.2 | 3452 - 9565 | 29.1 | 7.33 | 23.5 | 21.5 - 58.1 | 26.1 | 7.21 | 25.6 | 18.7 - 54.6 |
| 150 mg QW | Overall | 4672 | 1117 | 22.7 | 2557 - 8340 | 28.7 | 6.70 | 22.1 | 15.9 - 51.0 | 26.1 | 6.54 | 23.8 | 14.0 - 47.1 |
|  | US | 5200 | 759 | 14.5 | 4073 - 7557 | 31.8 | 4.59 | 14.4 | 25.0 - 46.0 | 29.3 | 4.36 | 14.8 | 22.8 - 42.9 |
|  | Non-US | 4532 | 1161 | 23.8 | 2557 - 8340 | 28.1 | 6.96 | 23.2 | 16.0 - 51.0 | 24.9 | 6.79 | 25.0 | 14.0 - 47.1 |
| 300 mg Q2W | Overall | 9366 | 2244 | 22.7 | 5122 - 16724 | 31.6 | 6.87 | 20.7 | 17.8 - 54.7 | 22.4 | 6.38 | 26.9 | 11.5 - 42.0 |
|  | US | 10425 | 1523 | 14.5 | 8164 - 15161 | 34.3 | 4.82 | 14.0 | 27.1 - 49.1 | 26.0 | 4.11 | 15.7 | 19.9 - 38.9 |
|  | Non-US | 9080 | 2332 | 23.8 | 5122 - 16724 | 31.0 | 7.14 | 21.7 | 17.8 - 54.7 | 21.0 | 6.61 | 28.4 | 11.5 - 42.0 |
