# Supplementary material for: Model-informed drug development of envafolimab, a subcutaneously injectable PD-L1 antibody, in patients with advanced solid tumors
Source: Oncologist. 2024 Jul 9;29(9):e1189–200. doi: 10.1093/oncolo/oyae102 (PMC11379657; doi:10.1093/oncolo/oyae102)
Supplement: oyae102_suppl_Supplementary_Figures [file oyae102_suppl_supplementary_figures.docx]

**Model-informed Drug Development of Envafolimab,** **a Subcutaneously Injectable PD-L1 Antibody, in Patients with Advanced Solid Tumors**

Cheng Cui^1, 2, †^, Jing Wang^1, 2, †^, Chunyang Wang^1, 2^, Ting Xu^3^, Lan Qin^4^, Shen Xiao^4^, John Gong^4^, Ling Song^1, 2, *^, Dongyang Liu^1, 2, *^

^1^ Drug Clinical Trial Center, Peking University Third Hospital, Beijing, China.

^2^ Institute of Medical Innovation and Research, Peking University Third Hospital, Beijing, China.

^3^ Alphamab Co., Ltd., Suzhou, China.

^4^ 3DMedicines Co., Ltd., Shanghai, China.

^†^Cheng Cui and Jing Wang contributed equally to this work and share first authorship.

^*^ Correspondence：

🖂 Dongyang Liu, liudongyang@vip.sina.com.

Drug Clinical Trial Center, Peking University Third Hospital, Beijing, 100191, China.

Tel: (010) - 82266456

^*^Co-Correspondence:

🖂 Ling Song, sl_onging@sina.cn.

Drug Clinical Trial Center, Peking University Third Hospital, Beijing, 100191, China.

Tel: (010) - 82266455

Key words: Envafolimab, PD-L1 antibody, subcutaneously injection, population pharmacokinetics, exposure-Response Analysis,

**CONFLICT OF INTEREST**

The authors declared no competing interests for this work.

**FUNDING**

This research was supported by Bill and Melinda Gates Foundation (INV- 007625).

Supplementary Figures

[Figure S1. Overall changes in target lesion size across the quartiles of C_min,1_, C_avg,1_, AU_Cinf,1_, and C_min,overall_. 3](#_Toc140927219)

[Figure S2. KM curves for DOR by median stratified C_min,1_, C_avg,1_, AUC_inf,1_, and C_min,overall_. 4](#_Toc140927220)

[Figure S3 Distribution of incidence and severity of top 4 AECI (increased AST, increased ALT, increased blood bilirubin, and rash) across C_max,1_ quartile 5](#_Toc140927221)

**
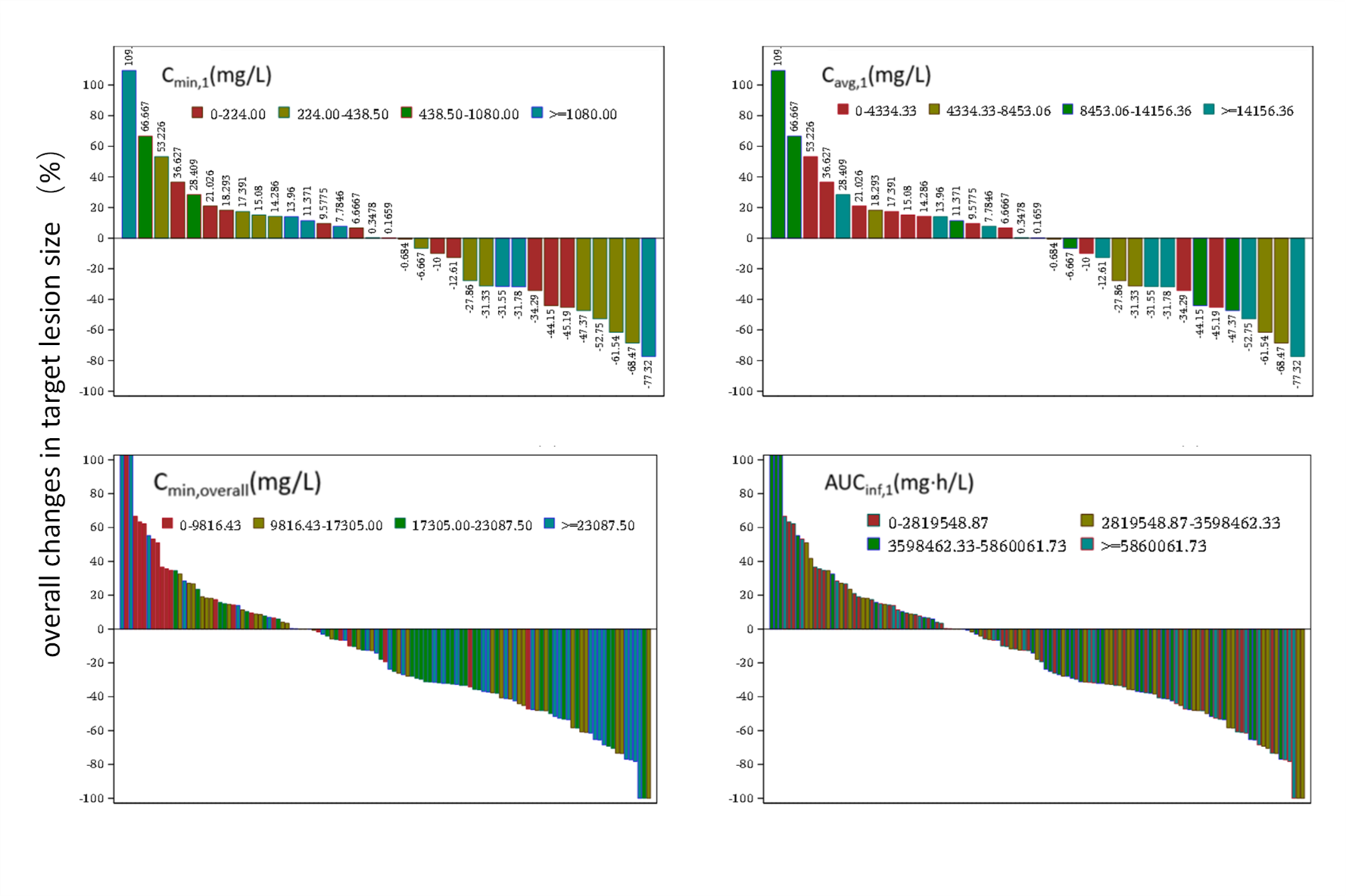
**

# Figure S1. Overall changes in target lesion size across the quartiles of C_min,1_, C_avg,1_, AU_Cinf,1_, and C_min,overall_.

# Figure S2. KM curves for DOR by median stratified C_min,1_, C_avg,1_, AUC_inf,1_, and C_min,overall_.

# Figure S3 Distribution of incidence and severity of top 4 AECI (increased AST, increased ALT, increased blood bilirubin, and rash) across C_max,1_ quartile

No AECI was reported in the third quartile for increased ALT, increased AST, and increased blood bilirubin
